# Pyrexia of Unknown Origin Caused by Non-Hodgkin’s Lymphoma: A Diagnostic Challenge for Clinicians

**DOI:** 10.7759/cureus.56742

**Published:** 2024-03-22

**Authors:** Hassan Hussain, Chaminda Janaka, Anne Sonali Rodrigo, Manojkumar Krishnan, Azra Hassan

**Affiliations:** 1 General Medicine, Sri Jayawardenepura General Hospital, Colombo, LKA; 2 Internal Medicine, Sri Jayawardenepura General Hospital, Colombo, LKA; 3 Pathology, Sri Jayawardenepura General Hospital, Colombo, LKA; 4 Medicine, Sri Jayawardenepura general Hospital, Colombo, LKA; 5 Oncology, District General Hospital Vavuniya, Colombo, LKA

**Keywords:** pyrexia of unknown origin, b lymphocytes, lymphoproliferative disorders, lymphadenopathy, non-hodgkin’s lymphoma

## Abstract

Non-Hodgkin’s lymphomas (NHLs) are a group of lymphoproliferative diseases that originate from different cell types, namely B cells, T cells, or natural killer cells. Herein, we report the case of a 69-year-old male patient who presented with a gradual-onset, intermittent, low-grade fever for four months and a right-sided neck lump for two months. On examination, a right-sided enlarged lymph node sized 1 × 1 cm^2^ was noted, which was mobile, hard in consistency, and non-tender. No other lymphadenopathy was noted in other parts of the body. Imaging and biochemical studies done at the initial stages did not reveal features in favor of any lymphoproliferative disorders, and the two lymph node biopsies done two weeks apart were inconclusive as well. An extended panel of investigations was done in view of excluding other infective and inflammatory pathologies, which was negative, making the patient undiagnosed of any disease state despite being symptomatic for four months. Finally, the third lymph node biopsy tested positive, which paved the way for the diagnosis of NHL. This diagnosis underlines the importance of this case. Following the diagnosis, the patient was initiated on a few chemotherapeutic agents, after which a mild symptomatic improvement was achieved.

## Introduction

Tumors of the lymphoid system can be broadly classified as Hodgkin’s lymphoma (HL) and non-Hodgkin’s lymphoma (NHL) [1]. NHL is a malignancy that has a wide range of presentations. It most commonly involves the lymph nodes and presents with generalized lymphadenopathy, yet there can be extranodal involvement as well. The extranodal sites include the stomach, lungs, and skin. The incidence of NHL has increased over the years, and among all malignant neoplasms, NHL usually presents in the 6th decade of life, with a preponderance among men [1]. 

NHL is characterized by abnormal cell clone proliferation, either involving T cells, B cells, or both cell types. Most of the NHLs originate from B cells, and the majority of patients express the CD20 antigen [2,3]. Considering the tumor activity, some of the NHLs are indolent malignancies with a lower tumor grade, while some NHLs are very aggressive [1]. NHLs are usually present at the age of 42, and the prevalence of the disease increases with age [4]. The most common histological type is diffuse large B-cell lymphoma. Various imaging modalities, including computed tomography (CT), magnetic resonance imaging (MRI), and positron emission tomography (PET), are valuable in finding nodal and extranodal sites of involvement, which can be further analyzed by tissue biopsies as well. Immunohistochemistry studies help in evaluating the positivity of CD20 and Ki67 [1].

## Case presentation

A 69-year-old retired teacher from Western Province, Sri Lanka, presented with a gradual-onset, intermittent, low-grade fever for four months. The fever was associated with chills, but not rigors, and was markedly noted in the evening and early hours of the morning every day. The fever slightly responded to simple analgesics but recurred from time to time. The patient reported a history of intermittent episodes of dry cough with whitish sputum for the same duration but no history of shortness of breath, pleuritic chest pain, or hemoptysis. He had no contact history of tuberculosis either. He denied having any history of headache, trauma to the head, or altered level of consciousness, as well as visual disturbances or photophobia. 

There was no history of recurrent vomiting or abdominal pain. The patient reported no changes in his bowel motions. He complained of a few lower urinary tract symptoms, such as urgency, increased frequency, and nocturia, for about six months and was on oral tamsulosin at 0.4 µm daily at the time of presentation. He denied a history of girdle pain or weakness in any part of the body. There was no history of joint pains, alopecia, or skin rashes, and the patient did not have a history of significant weight loss, loss of appetite, or night sweats.

The patient traveled to India in 2019 on a religious pilgrimage and had been there for 20 days. However, no history of illnesses or hospital admissions around the time was noted. He had no history of exposure to animals or poultry and no history of consuming unpasteurized milk.

The patient reported noting a lump on the right side of his neck about two months ago. Initially, it was about 0.5 × 0.5 cm^2^ in size, and no skin changes like erythema or discharges were noted from the lump. Since the patient did not see any significant increase in the size of the lump over the past two months, he did not seek medical advice. His past medical history was insignificant, with no history of valvular heart diseases. He underwent a left-sided laser lithotripsy procedure nine months ago due to a left-sided renal calculus. He denied a family history of any chronic medical conditions or malignancies. He was a non-smoker and a teetotaler with no history of illicit drug abuse. He denied having risky sexual behaviors and was not allergic to any substance, drug, or food item.

The patient, who lived with his wife and daughter, reported having good family support and financial stability. The patient was an averagely built male whose appearance was consistent with his chronological age. He had a body weight of 43 kg, a height of 171 cm, and a body mass index (BMI) of 14.7. He was afebrile at the time of admission. There was no conjunctival pallor, plethora, or icterus. On the right side, there was an enlarged lymph node, which was around 1 × 1 cm^2^ in size. It was mobile, hard on consistency, and non-tender. No other part of the body showed any signs of lymphadenopathy. No enlarged thyroid gland was noted. There were no skin rashes, and no peripheral stigmata of infective endocarditis was present. Ankle edema was not noted. 

The patient had a pulse rate of 80 beats per minute, which was regular and normal in volume and character. Peripheral pulses were present, and there were no delays. His blood pressure was 120/80 mmHg. The cardiovascular system examination was unremarkable, with no murmurs. He had a respiratory rate of 12 per minute with bilateral equal air entry in all zones and vesicular breathing. No added sounds like crepitations or rhonchi were noted. Abdominal examination was unremarkable, with no organomegaly, palpable masses, or free fluid. He did not show any signs of meningism, and the fundoscopic examination was unremarkable, with absent Roth spots, toxoplasma chorioretinitis, choroid tubercles, or cystoid bodies. Cranial nerve examination was normal, and no features of peripheral neuropathy were present.

To arrive at a diagnosis, serial investigations were performed, which have been summarized in Table 1. Despite extensive evaluation, no diagnosis for the patient’s presentation could be elicited, and he was discharged after 10 days of admission to be reviewed with the right-sided lymph node biopsy report. He was not initiated on any antibiotics during this admission.

**Table 1:** Investigations ANA: anti-nuclear antibody; CEA: carcinoembryonic antigen; ESR: erythrocyte sedimentation rate; NHL: non-Hodgkin’s lymphoma; PSA: prostate-specific antigen; T4: thyroxine; TIBC: total iron binding capacity; TSH: thyroid-stimulating hormone.

Investigations		Values						Reference range
		1^st^ admission	2^nd^ admission					
		D1	D27	D33	D35	D38	D41	
Full blood count	White cell count (*10^3^)	6.74	6.60	6.63	6.07	8.54	7.59	4–11
	Neutrophils (%)	62	3.8	33	48	61	62	50–70
	Lymphocytes (%)	22	1.78	33	34	26	25	20–40
	Hemoglobin (g/dL)	11.8	9.8	11.9	10	10.8	10.5	12–14
	Platelets (*10^3^/µL)	411	290	329	296	307	370	150–400
Liver biochemistry	Alanine transaminases (U/L)	144	-	-	-	-	24	<40
	Aspartate transaminases (U/L)	22	-	-	-	-	33	0–37
	Alkaline phosphatase (U/L)	144	-	-	-	-	164	30–120
	Total bilirubin (mg/dL)	0.4	-	-	-	-	0.5	0.2–1.1
	Serum albumin (g/dL)	-	-	-	-	-	3.1	3.4–5.4
	Serum globulin	-	-	-	-	-	3.9	2–4
Inflammatory markers	C-reactive protein (mg/L)	65	49	91	25	32	76	<5
	Erythrocyte sedimentation rate (mm in 1^st^ hour)	76	88	90	-	-	67	<20
	Procalcitonin (ng/mL)	0.1	-	-	-	-	-	<0.1
Serum electrolytes	Serum sodium (mmol/L)	141	-	-	-	-	-	135–145
	Serum potassium (mmol/L)	3.7	-	-	-	-	-	3.5–5.5
	Serum total calcium (mg/dL)	-	-	-	8.40	-	-	8.5–10.5
	Adjusted calcium (mg/dL)	-	-	-	8.88	-	-	8.6–10
Renal function tests	Serum creatinine (µmol/L)	-	69	-	-	-	-	65–104
Other investigations	D-dimers (ng/ml)	-	-	-	-	1982	-	<550
	Serum lactate dehydrogenase (U/L)	187	-	-	-	-	205	<330
Tumor markers	CEA (ng/ml)	-	-	-	-	-	2.4	0–5
	Alpha fetoprotein (ng/ml)	-	-	-	-	-	1.49	0–8.5
	CA 19-9 (U/ml)	-	-	-	-	-	4	<35
Iron studies	Serum iron (µmol/L)	2.9						10–30
	Total iron binding capacity (mcg/dL)	27						240–450
	Transferrin saturation (%)	10.3						15–50
	Serum ferritin (ng/mL)	250						20–250
ANA test		Negative						
Rheumatoid factor		Negative						
Thyroid profile	Serum TSH (µIU/mL)	0.588						0.35–5.5
	Free T4 (ng/dL)	1.06						0.7–1.48
PSA (ng/mL)		2.4						<4
Blood picture		Mild anemia with left shift of neutrophils with toxic changes suggestive of infective or inflammatory process.						
Right-cervical lymph node biopsy-1 (1^st^ admission)		Appearances are compatible with chronic sialadenitis of submandibular gland. No evidence of a lymph node.						
Right-cervical lymph node biopsy-2 (2^nd^ admission)		Inconclusive viral infection is most likely						
Right-cervical lymph node biopsy-3 (2^nd^ admission)		Left cervical lymph node level 2 showing features of high-grade non-Hodgkin lymphoma of B lineage.						
Bone marrow biopsy		Reactive marrow with granulocytic hyperplasia with mildly increased monocytes/macrophage activity evidence of mild iron deficiency noted no evidence of leukemia/lymphoma/myeloma or non-hemopoietic cell infiltration trephine biopsy-consistent with bone marrow aspiration findings.						
Schirmer's test		Negative						
Serum protein electrophoresis		Reduced albumin. But no abnormal bands.						

The patient was readmitted 27 days after the initial admission for further investigation of a fever. At the time, he had the biopsy report from the previous admission, which showed evidence of chronic sialadenitis of the submandibular gland with no evidence of lymphadenopathy [1]. Hence, the diagnosis was inconclusive, and serial imaging studies were performed. Furthermore, infective screening (Table 2) as well as imaging studies (Table 3) were performed as well.

**Table 2:** Infective screening Ab: antibodies; CSF: cerebrospinal fluid; EBV: Epstein-Barr virus; HBsAg: hepatitis B surface antigen; HCV Ab: hepatitis C antibody; HIV: human immunodeficiency virus; RDT: rapid diagnostic tests; TB: tuberculosis; VDRL: venereal disease research laboratory test.

Infective screening	Results
Urine full report	Normal (repeated 3 times)
Urine culture	No growth (repeated 3 times)
Blood culture	No growth (repeated 3 times)
Sputum culture	No growth
Sputum GeneXpert	Negative
Stool for microscopy	No parasites, amoeba, cyst, or ova seen
CSF analysis	Appearance - colorless/clear glucose – 45 mg/dL (50-85) protein – 57 mg/dl (15-45) white cell count – nil red cells - 168
CSF culture	No growth
TB culture of bone marrow aspirate	Not isolated after 2 weeks of growth
Sputum acid fast bacilli	3 samples negative
Malarial parasite (microscopy and RDT)	Negative
HIV 1 and 2 antibodies	Negative
Mantoux test	Negative
Venereal diseases laboratory test	Non-reactive
Urine for acid fast bacilli	Not seen
Filaria antibody test	Negative
Brucella antibody	Negative
Hepatitis B surface antigen	Negative
Hepatitis C antibody	Negative
Salmonella typhi O,H and Para typhi antigens	Negative
Melioidosis antibodies	Negative
Epstein Barr virus antibodies	Negative
Typhoid antibodies	Negative

**Table 3:** Imaging studies 2D Echo: two-dimensional echocardiography; CECT: contrast-enhanced computed tomography; TEE: transesophageal echocardiography; USS KUBP: ultrasound scan of kidney, ureter, bladder, and prostate.

Imaging studies	Results
Upper gastrointestinal endoscopy	Normal
Lower gastrointestinal endoscopy	Normal
Trans esophageal echocardiography	No evidence of infectious endocarditis
2D echocardiography	Normal. Did not have vegetation or shunts
USS KUBP	Prostatomegaly with no significant post-voidal volume.
CECT chest, abdomen, and pelvis	Heterogenous-enhancing area in anterior mediastinum most likely residual thymic tissues. Few prominent bilateral cervical lymph nodes noted bilateral non-obstructing renal calculi simple liver cyst in segment v11 of liver.
Venous doppler study of bilateral lower limbs	No features of deep vein thrombosis
Ultrasound scan of neck	Features suggestive of chronic submandibular sialadenitis. Bilateral cervical lymphadenopathy with possible necrotic lymph nodes at level 2.

**Figure 1:**
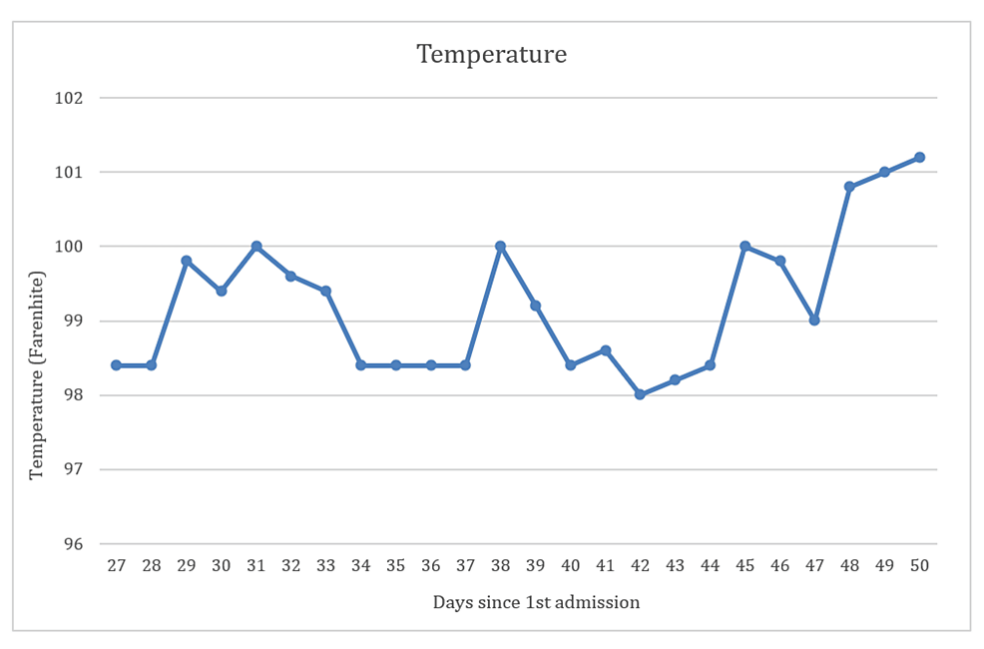
Body temperature chart plotted against the days since admission

The patient was initiated on antibiotics during this admission, which included an intravenous combination of cefuroxime, ceftriaxone, piperacillin, and tazobactam, along with meropenem and doxycycline. Despite antibiotic administration, his fever continued with a slight variation in severity. The pattern of fever is shown in Figure 1.

**Figure 2:**
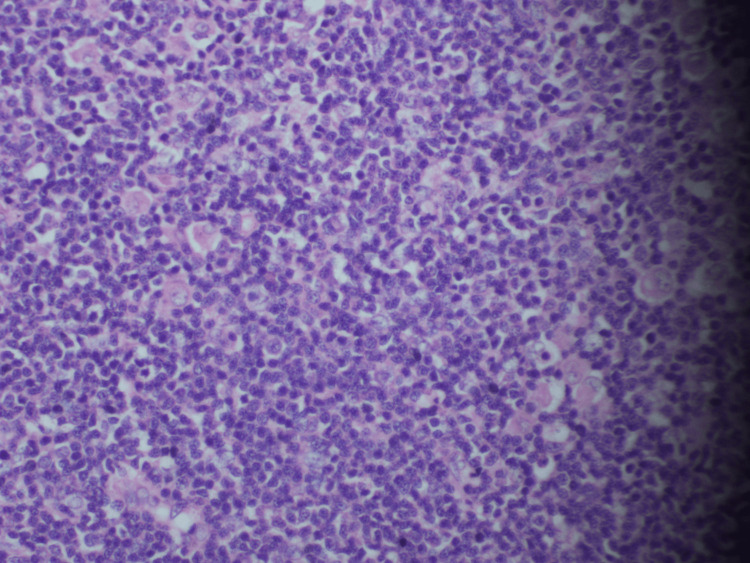
Microscopic view of the left-side level 2 cervical lymph node

Hematoxylin and eosin (H&E) staining of the left-sided level 2 cervical lymph node showed atypical lymphoid cells with enlarged nuclei, clumped chromatin, and moderate-to-scant eosinophilic cytoplasm. We observed mitoses without any signs of tumor necrosis. These features are compatible with diffuse large B-cell lymphoma (Figure 2).

**Figure 3:**
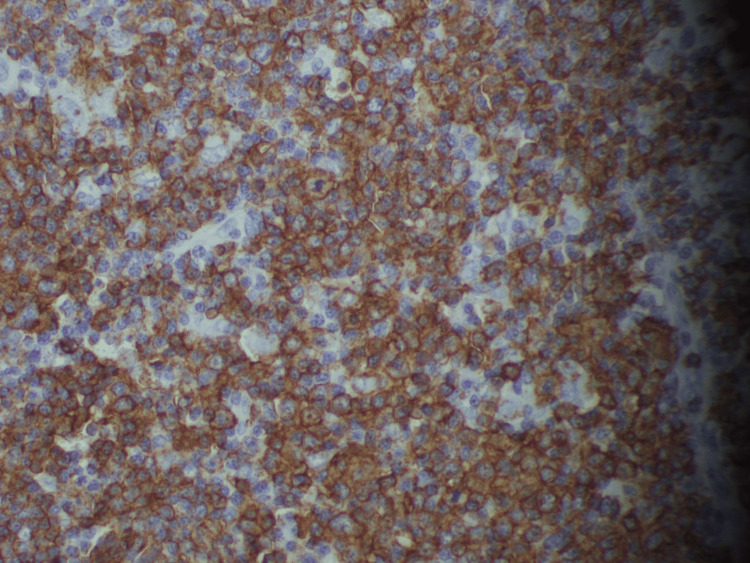
Immunohistochemistry of the left-sided level 2 cervical lymph node showing diffuse cytoplasmic positivity for CD20 and few background cells showing cytoplasmic positivity for CD3

Although almost all the investigations were normal at the initial stages, the third lymph node biopsy performed during the second admission showed evidence of diffuse large B-cell lymphoma with germinal center B cell type (Figure 3) [1]. Hence, an NHL diagnosis was confirmed, and the patient was initiated on chemotherapeutic management with rituximab, cyclophosphamide, doxorubicin, and vincristine, after which a symptomatic improvement in his clinical state was noted.

## Discussion

Malignancies of the lymphoreticular system are sometimes collectively called lymphomas. These malignancies stem from lymphatic tissue and may extend to an extra-nodal site (NHL) or transform into non-tender masses in the lymph node region (HL). At the latter stages, they may spread to various other lymph node groups and may involve the bone marrow [1]. Based on the World Health Organization’s (WHO) modified version of the Revised European-American Lymphoma classification, lymphoid malignancies can be classified into three sub-categories, namely B-cell lymphoma, T-cell or natural killer cell lymphoma, and HL [1]. 

Among head and neck malignancies, the NHL has a special place, and Waldeyer’s ring is the most common site of origin for these NHLs [1,5]. Most head and neck NHLs have cervical nodal involvement as well. This was seen in this case as well. Paranasal sinuses, orbits, salivary glands, and the nose are among the other sites in the head and neck region that are commonly involved in NHLs [5]. Considering the clinical course of the disease, NHLs have varying clinical presentations and heterogeneous morphological appearances. A wide variation in response to treatment is also seen in NHLs. The cellular variation of NHLs depends on at which stage of development the cells are arrested, their grade of anti-apoptotic abnormality, etc. [1].

Based on cell maturation, NHLs are further classified as immature and mature variants. Based on the cell of origin, they are classified as B cells, T cells, or natural killer cell types [1]. Immunohistochemistry markers vary for each type; while CD20 and CD79a are used as markers for B-cell tumors, CD3 and CD5 are used as markers for T-cell tumors [6]. Immunohistochemistry markers are important in determining the specific lineages and the stage of development of the lymphoma. In this case, immunohistochemistry studies revealed positivity for CD20 and BCL2, and stains were negative for CD3 and CD10. Hence, it was identified as a diffuse B-cell lymphoma. The tumor grade was identified as a high-grade tumor, as the Ki67 index was 71%. 

Primary lymphomas are common in males in their fifth and sixth decades of life. Considering the presentation of our case, the age group and gender of the patient matched the classical presentation of a patient with NHL (a male patient aged 69 years). In terms of geographical location, NHLs are more common in developed countries than in developing nations. Since this patient was from Sri Lanka, it did not align with the above finding. 

The diagnosis of NHL is based on imaging studies, histological evaluation, and immunohistochemistry. In diffuse large B-cell lymphoma, the most sensitive investigation is a PET scan [7]. It has been shown to have more sensitivity than a bone marrow biopsy [8]. This was evident in this case because the bone marrow biopsy done in this patient did not reveal evidence of any lymphoproliferative disorders, and it led to the diagnosis being missed at the initial stage. A PET scan could not be performed on this patient due to financial constraints. However, a PET scan is not foolproof, as there would still be a chance of missed diagnosis in the case of low-volume diffused lesions [7]. But this outweighs the benefits of a PET scan, which enables the diagnosis of the majority of lesions at the first instance itself. Apart from diagnosis, a PET scan is useful in monitoring response to treatment and for restaging the disease following treatment in both HL and NHL. 

The Ann Arbor staging system is used for the management of NHLs affecting the head and neck region (Table 4) [1]. Low-grade tumors are usually managed with radiotherapy alone, while patients with high-grade lymphoma with a disseminated clinical picture are treated with a combination of chemotherapy and radiation therapy. This patient was initiated on a chemotherapeutic regime alone, and he showed a good response despite the NHL being a high-grade tumor. Although there is a place for surgical resection for isolated lesions [1], in most cases, surgical enucleation is combined with radiotherapy and chemotherapy for better outcomes.

**Table 4:** Ann arbor staging system for NHLs NHL: non-Hodgkin’s lymphoma.

Stage	Features
Stage 1	Involvement of a single lymph node region or lymphoid structure
Stage 2	Involvement of 2 or more lymph node regions on the same side of the diaphragm
Stage 3	Involvement of lymph regions or structures on both sides of the diaphragm
Stage 4	Involvement of extranodal site beyond that is designated in E
For all stages	
A	No symptoms
B	Fever (38 °C), drenching sweats, weight loss (10% body weight over 6 months)
For stages 1 to 3	
E	Involvement of a single, extra nodal site contiguous or proximal to known nodal site

Diffuse large B-cell lymphoma usually has an aggressive disease progression, with 30-60% of tumors being curable with intensive chemotherapy coupled with rituximab, which is why it was initiated in this patient [9]. R-CHOP (rituximab, cyclophosphamide, hydroxydaunorubicin, oncovin, and prednisone) is the classic protocol initiated in patients with aggressive NHL. It comprises six to seven cycles of rituximab, cyclophosphamide, doxorubicin, vincristine, and prednisolone [9].

## Conclusions

NHLs can have varying presentations, and the initial investigations can be negative despite the patient harboring the disease. Hence, this case emphasizes the importance of proper examination, the value of performing serial investigations, and, if clinically indicated, the necessity of performing repeated histological biopsies to arrive at a proper tissue diagnosis. Histological diagnosis is vital and paramount for successful patient treatment and for predicting the disease outcome following treatment.
